# Alternative drugs go global: possible lead and/ or mercury intoxication from imported natural health products and a need for scientifically evaluated poisoning monitoring from environmental exposures

**DOI:** 10.1186/s12995-016-0139-0

**Published:** 2016-11-08

**Authors:** Lygia Therese Budnik, Xaver Baur, Volker Harth, Axel Hahn

**Affiliations:** 1 Institute for Occupational and Maritime Medicine (ZfAM), University Medical Center Hamburg-Eppendorf, Hamburg, Germany; 2 Charite Institute for Occupational Medicine (CIOM), Charite-University Medicine, Berlin, Germany; 3 Federal Institute for Risk Assessment (BfR), Berlin, Germany; 4 European Society for Environmental and Occupational Medicine (EOM), Berlin, Germany; 5 Occupational Toxicology and Immunology, Institute for Occupational and Maritime Medicine (ZfAM), University Medical Center Hamburg-Eppendorf, Marckmannstrasse 129 B, 20539 Hamburg, Germany

**Keywords:** Natural health products, Dietary supplements, Heavy metal intoxication, Lead, Mercury, Monitoring, Prevention measures

## Abstract

**Background:**

With increases in globalization, cultural remedies from Chinese, Ayurvedic, Arab and other traditions have become more available to international consumers, offering unfamiliar “Natural Health Products” (NHP), used as alternative medicine or supplementary medicine.

Contamination with toxic ingredients including lead, mercury, arsenic, and other toxic elements has been documented in several of these products from various parts of the globe, particularly from some parts of Asia and the Orient.

**Findings:**

We have been following this development in the last 6 years and have analyzed *n* = 20 such products (60 analyses) from patients with intoxication symptoms in a pilot study, showing alarming high concentrations of mercury and/or lead (the first one in “therapeutic” doses). 82 % of the studied NHP contained lead concentrations above the EU limit for dietary supplements. 62 % of the samples exceeded the limit values for mercury. Elevated blood lead and mercury levels in patients along with clinical intoxication symptoms corroborate the causal assumption of intoxication (s). We present one detailed clinical case report of severe lead and mercury intoxications and give an overview about blood concentration related symptoms and signs of *n* = 41 case reports of mercury intoxications of the German monitoring BfR-DocCenter.

**Conclusions:**

For NHP there is evidence on a distinct toxicological risk with alarming low awareness for a possible intoxication which prevents potentially life-saving diagnostic steps in affected cases. In many cases patients do not communicate the events to their physicians or the local health authority so that case reports (e.g. the BfR-DocCentre) are missing. Thus, there is an urgent need to raise awareness and to initiate more suitable monitory systems (e.g. National Monitoring of Poisonings) and control practice protecting the public.

## Background

### Important underestimated global health issue

With the globalization, Chinese, Ayurvedic, African or Arab complementary medicine became available to international consumers [1]. People worldwide are increasingly consuming diverse natural health products, NHP (in the EU dietary supplements) with estimated 600 million visits for users of alternative medicine [1]. Patients believe that the intake of these products is safe and unlike conventional medical therapies devoid of side effects, since they assume that NHP are prepared only out of herbs, animal and/or natural minerals. Notably some natural medicine therapy schools treat in accordance with the valid doctrine “*similis similibus curantur*” claiming that heavy metals can be used (in supposedly safe doses) for therapeutic detoxification. Case reports show, however, heavy metal intoxication in NHP consumers worldwide [2–5]. American Studies [5] found in 20 % (*n* = 197) of such products lead, mercury and arsenic in concentrations 100–10,000 times higher than the allowable limit values. Our goal was to analyze and evaluate such natural products sent to our laboratory for indicatory analysis in the course of suspected heavy metal intoxications.

Additionally, we aimed to compare the data with intoxication cases reported to the BfR-DocCenter and reevaluate the existing data based on well documented human reports [6]. Since new data have been recently published on complex effects of elementary lead and mercury on human health our goal was to reassess the current situation in Germany with regard to the possible intoxications to lead and mercury.

## Findings

### Pilot study

Our pilot study is case-based, non-randomized. We have included all patients with presumed heavy metal intoxication due to NHP within 6 years refereed to us from clinical departments (i.e. neurology) for a second diagnosis. Additionally, all cases reported to the BfR-DocCenter were analyzed representing the official statistics from Germany. Limitation: since this is a case based study, we are not aware on dark figures, i.e. of non-reported cases.

The samples were analyzed in the period 2009 to 2015. Product samples were extracted using microwave –assisted process [7–9] with PTFE-HT tubes, HTR -300/6 s dissociation with pressure-calibration system (PCS) from MLC (Mikrowellen Laborsysteme, Leuterbach, Germany). Material samples as well as blood and urine samples were quantified using atomic absorption spectrometer [10] with automatic sampler system (Thermo Fisher Scientific, Dual AA Spectrometer ICE 3500, Freiburg, Germany). For mercury the AA method with hybrid metal mercury enhancement system was applied and lead was measured using graphite oven with Zeeman background compensation; LOD values for lead were 0.01 μg/g and for mercury 0.02 μg/g dry mass. Pb and Hg were quantified based on the ratio of analyte to that of internal standard in peak hopping mode. Two bench quality control pools were analyzed along with the samples. Signal data were analyzed with data processing software (Thermo Fisher). The internal and external quality assessment scheme followed the rules of the German guideline for human biomonitoring with external quality assessment scheme from the German Society for Occupational and Environmental Medicine www.g-equas.de. The measured day to day precision was <15 % RSD The statistical analyses were performed with Prism version 6.00 for Windows, GraphPad Software, La Jolla, California, USA.

**Fig. 1:**
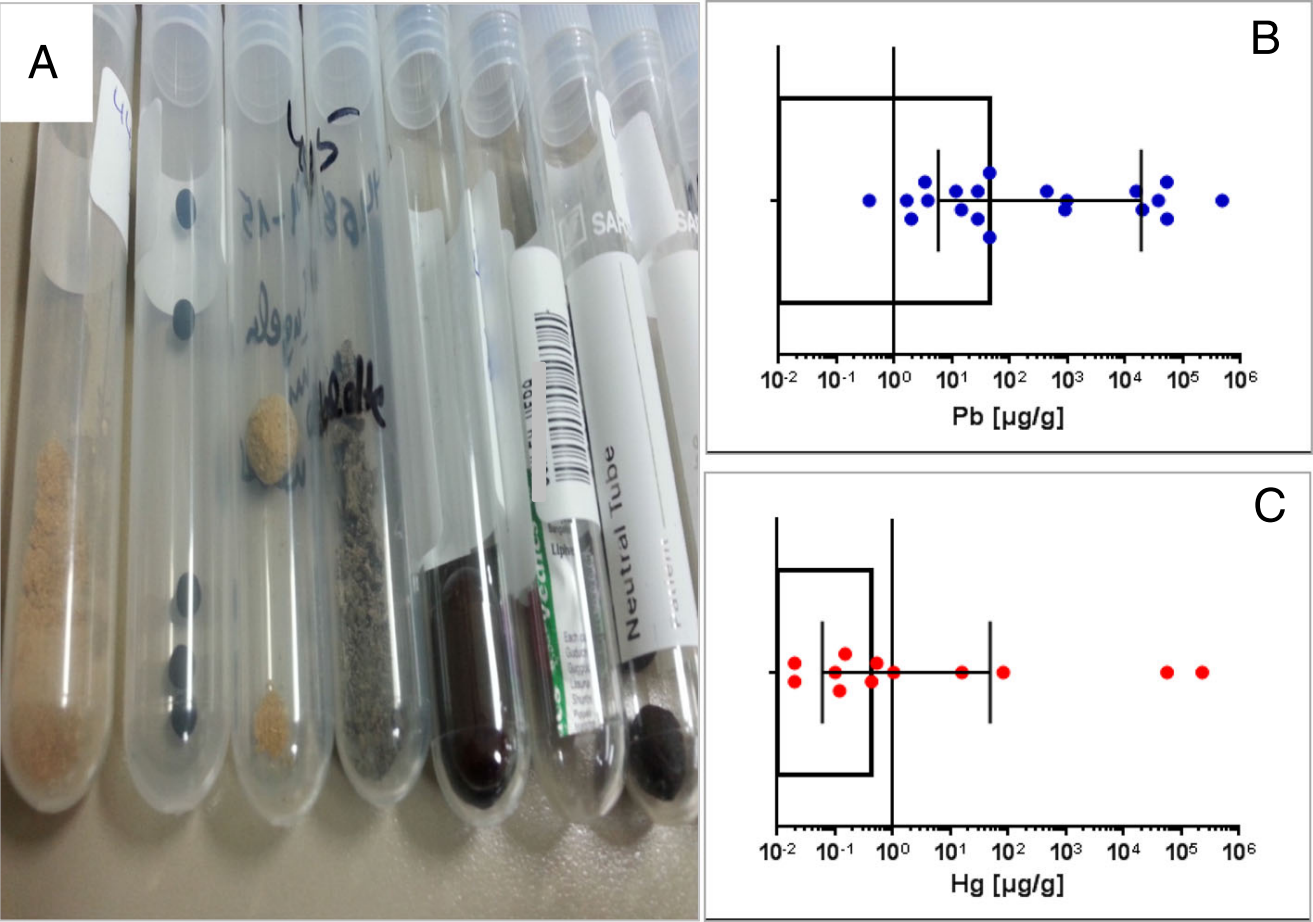
Lead and/or mercury contamination in natural health products (NHP). **a** NHP (in the EU dietary supplements, for oral exposure road) are sold as powder, globules (in different size or pressed into tablets). B/C. Scatter plot (with box showing median values with inter quartile range) of *n* = 20 NHP tested for lead (**b**) and mercury (**c**) contamination. Note: dots below LOD are not shown

Product analyses from patients with presumed intoxication due to NHP: symptoms included abdominal cramps, anemia, neuropathy and kidney failure (see below for a typical case presentation). 20 natural health products were measured (Pb, Hg, As content) that patients either brought from abroad or had ordered over the Internet (Fig. 1a). The remedies were analyzed by atomic absorption spectrometry (see above).

Eighty-two percent of the studied NHP contained lead concentrations above the EU limit (Table 1) for dietary supplements (3 μg/g). Average amounts of lead were: 35.80 μg/g, [3.59 to 17,760 μg/g for 25 % vs. 75 % percentile respectively; Median values were 45 μg/g]. 62 % of the samples exceeded the limit values for mercury (0.1 μg/g), average mercury amounts found were: 22.13 μg/g [0.06 to 46.6 μg/g for 25 % vs. 75 % percentile respectively; median: 0.43 μg/g] (Fig. 1b, c). Arsenic was detected in a single sample.

**Table 1:** Limit values

Heavy metal	Diagnostic analysis (biomonitoring parameter)	Reference values [μg/L]	Limit values [μg/L]	NHP^c^ allowable limit values [μg/g]
Lead	total blood lead	60^a^ 42^b^	150/250^d^	3
	total urine lead	- 2.3^b^	-	
Mercury	total blood mercury	2^a^ 4.6^b^	5	0.1
	urinary total mercury	1.0^a^ 3.3^b^	7	
Arsenic	total blood arsenic	-	-	n.d.
	urinary total arsenic	15^a^ 66^b^	n.d.	

Limit values refer to adults >20 y (German Human Biomonitoring values). Reference values refer to adults: National report on human exposure to environmental chemicals, showing 95^th^ percentile values: ^a^Germany (https://www.umweltbundesamt.de/themen/gesundheit/kommissionen-arbeitsgruppen/kommission-human-biomonitoring/stellungnahmen-der-kommission-human-biomonitoring); ^b^US (http://www.cdc.gov/exposurereport/pdf/fourthreport.pdf); ^c^EU allowable limit values for dietary supplements

n.d. not determined; ^d^the values are not valid after 2013 (are currently being re-evaluated)

### Case report: patient with lead and mercury intoxication

Patient, male, 31 with BMI slightly below normal, non-smoker, was referred to the neurological department of the university clinic with severe peripheral poly neuropathy and sensory motor symptoms with neuropathic pain. The patient was in good general state of health until approximately 3 weeks before hospital admission; he spent his holiday in Himalaya region and came back with headaches and fatigue. He was taking pain medication without any relieve; his routine blood values were normal. He claimed to take no further medications. Since poly neuropathy and fatigue could be caused by pesticides or other poisoning, i.e. heavy metals, we have been consulted for taking a detailed exposure history. While in the clinic, 3 different NHPs were found in form of globules, (a, b, c for morning, lunch time and evening respectively), which he imported from his trip to Asia and ingested 3 times a day against stress. We have analyzed these 3 NHPs and found: 45 μg/g, 53,000 μg/g and 28 μg/g lead (for morning, midday and evening globules, respectively) and additionally 15.72 μg/g mercury in the “evening globules”. Since, his blood metal levels were: 340 μg/L Pb and 15 μg/L Hg a diagnosis of heavy metal intoxication was made. Slowly occurring clinical recovery after starting chelation therapy corroborated with the causal assumption proposed. He was released for further consultancy to his family physician. The administrated treatment and the improvement of his status corroborate lead and mercury intoxication.

### Lead intoxication cases: symptoms and signs organ related

While looking closer for reported cases to the BfR-DocCenter [11], we found one similar case reported to this national poisoning report system. The case report describe female patient with neurological symptoms, radial paralysis and anemia with 880 μg/L blood lead levels after consuming lead containing NHP (with 50,400 μg lead/g) for several weeks. CDC-NIOSH funded the ABLES (adult blood lead epidemiology & surveillance) program and conducted adult blood lead levels (BLL) surveillance [12]. In 2010, ABLES program reported 31,081 adults with BLL ≥ 100 μg/L [13]. In the United States, when the exposure source is known, approximately 95 % of BLL ≥ 250 μg/L in adults are work-related (battery manufacturing, lead and zinc ore mining, painting and paper handling industries). The National Toxicology Program data have shown that low lead exposure causes acute and chronic adverse effects in multiple organ systems [14]. Beside, well known symptoms like abdominal cramps, anemia [15] also confusion, neuropathy, encephalopathy or paralyses may occur. Evidence indicates that lead exposure at low doses can lead to adverse cardiovascular and kidney effects [16] (tubular dysfunction, increased glomerular filtration rate), cognitive dysfunction and adverse reproductive outcomes. Decreased renal function is associated with BLL at 50 μg/L or lower and increased risk of hypertension and essential tremor at BLL below 100 μg/L [14]. Lead can also constrain the growth of long bones in the childhood and increase susceptibility to osteoporosis later in life [17, 18].

### Mercury intoxication cases: symptoms and signs blood level related

Unlike for lead we found 41 reported cases (BfR-DocCenter-Database) with exposure to elemental and inorganic Hg (Table 2), which had been conditioned scientifically before [19]. The cases were evaluated and recorded by the BfR expert-judgment [20]. The evaluated cases were assessed with regard to the dose, standardized symptoms and signs. and categorized for health impairment following the Poison Severity Score (PSS). The causality (exposure vs. symptoms/signs) was assessed by the BfR-standard “Three-Level-Model”. Symptoms and signs were carefully assessed and aggregated in concentration-related sub- and top groups to get Hg-specific impairment pattern beside the standard PSS assessment. Additional clinical data were evaluated in terms of the different dose-response relationship among the various groups (Table 2﻿). Nearly half the cases in which all grades of PSS (see [20] for more details) are represented have Hg blood concentration between 15 and 40 μg/L, which is only slightly above the HBM-II (German Human Biomonitoring limit values) value.

**Table 2:** Cases of elemental and inorganic mercury exposure (*n* = 41) Symptoms and signs organ related vs. Hg blood concentraton

Symptoms/signs		Mercury concentration in blood		
Organ top groups	Sub-groups	≤25 μg/L	≤ 300 μg/L	≤ 3000 μg/L
Gastrointestinal		X	x	
	abdominal pain	X	x	
Neurological		X		x
	disorders of consciousness		x	
	neuropathy	X	x	
	headache			
	sweating disturbance	X	x	
	sleeping disturbance	X	x	
	memory disturbance		x	
Neuro-psychosocial		X	x	x
	personality disorder	X	x	
	depression		x	
	weakness	X	x	
	lost of weight	X	x	
	thirst		x	
Muscle/ skeleton/teeth		X	x	
	muscle pain	X	x	
	teeth disorders		x	
Cardio- vascular/blood		X	x	
	cardiomyopathy		x	
	body temp. elevated	X	x	
	panmyelopathy	X		
	blood count disorders	X	x	
Respiratory		X	x	
	pneumo thorax		x	
	hypoventilation	X	x	
	acidosis		x	
Liver/Kidney		X		
	hepatomegaly		x	
	kidney disorders	X	x	
Skin				x
	necrosis	X	x	
	alopecia	X	x	
Eye				
	eye disorders	X	x	

41 cases with elemental and inorganic Hg, reported to the BfR DocCenter were analysed. The cases were evaluated by the BfR expert judgement and were assesses regarding dose against the BfR standardised symptoms and signs, followed by the Poison Severity Score. The causality exposure vs symptoms was assessed by the BfR “Three-Level-Model”

## Conclusions and future perspectives

Serious heavy metal intoxications from imported NHP products occurred in Germany so far only in individual cases; in comparison the rate in the US and other Asian countries is much higher. About the estimated number of unreported cases (or unrecognized cases) there are no studies. But the lead and mercury concentrations in our analyzed natural health products are alarmingly high; so the ingestion of larger amounts might be harmful and with the danger of heavy metal intoxication. Metallic mercury is naturally occurring in the environment and is often extracted from cinnabar ore. It is used in products such as thermometers, electric switches and dental fillings, as well as for the production of caustic soda and chlorine gas. Exposure to mercury often results from consumption of contaminated food sources, breast milk or cosmetic products [10]. Lead occurs naturally in the environment and has been mined for use in a variety of products, including paint, dyes, ceramic glazes, pesticides, ammunition, pipes, weights, cable covers, car batteries and sheets used for protection from radiation. Lead, is one of the nine targeted risk factors (4 % contribution in 2014) for estimated burden of diseases in Europe and recalls for 100–900 DALYs per million people in central Europe [21].

In many countries, even in Germany, no comprehensive nutria vigilance- or poisoning monitoring system exists, from which the application of natural health products and the consequent intoxication can be estimated [11]. There is also an urgent need for comprehensive scientifically evaluated studies based on efficient national monitoring to protect the consumer from heavy metal intoxications. There are no comparable surveillance systems like the US ABLES program for lead- and no surveillance systems for mercury exposures allowing any comparisons. Exposure to lead and mercury from environmental sources remains an overlooked and serious public health risk.
